# Mechanical Intelligence (MI): A Bioinspired Concept for Transforming Engineering Design

**DOI:** 10.1002/advs.202203783

**Published:** 2022-09-14

**Authors:** Ali Khaheshi, Hamed Rajabi

**Affiliations:** ^1^ Division of Mechanical Engineering and Design School of Engineering London South Bank University London SE1 0AA UK

**Keywords:** adaptive structures, bioinspired designs, biomimetics, shape morphing, variable stiffness

## Abstract

Despite significant scientific advances in the past decades, most structures around us are static and ironically outdated from a technological perspective. Static structures have limited efficiency and durability and typically perform only a single task. Adaptive structures, in contrast, adjust to different conditions, tasks, and functions. They not only offer multi‐functionality but also enhanced efficiency and durability. Despite their obvious advantages over conventional structures, adaptive structures have only been limitedly used in everyday life applications. This is because adaptive structures often require sophisticated sensing, feedback, and controls, which make them costly, heavy, and complicated. To overcome this problem, here the concept of Mechanical Intelligence (MI) is introduced to promote the development of engineering systems that adapt to circumstances in a passive‐automatic way. MI will offer a new paradigm for designing structural components with superior capabilities. As adaptability has been rewarded throughout evolution, nature provides one of the richest sources of inspiration for developing adaptive structures. MI explores nature‐inspired mechanisms for automatic adaptability and translates them into a new generation of mechanically intelligent components. MI structures, presenting widely accessible bioinspired solutions for adaptability, will facilitate more inclusive and sustainable industrial development, reflective of Goal 9 of the 2030 Agenda for Sustainable Development.

## Graphical abstract

Mechanical Intelligence (MI) offers a new paradigm for designing next generation of adaptive structures with superior capabilities. It explores nature‐inspired mechanisms for automatic adaptability and applies them to engineering structures that can adapt to applied loads without active actuation. Offering enhanced efficiency, durability, and multi‐functionality, MI structures are more sustainable and less costly compared to the existing solutions.

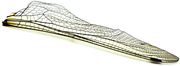

*“The wings are in effect “smart” aerofoils, combining remote and automatic shape control in ways which seem to occur nowhere else in nature or in technology”* – Robin J Wootton^[^ ^1^ ^]^

Imagine a dynamic world, where structures change their shape and properties to adapt to different needs; buildings that expand to allow more people in, bridges that morph to open pathways for large vehicles, columns that deform to withstand higher loads, and cars that strengthen their body to protect passengers in crashes. These are scenarios where conventional structures fail to function, as they cannot adapt to continuously changing conditions. This is true for most structures around us that are static; their shapes and properties are set to meet functional demands for limited tasks that once defined are not expected to change during operational lifetime.

Modern engineering applications require structures that have variable shapes and/or stiffness levels, and therefore can adapt to different conditions, tasks, and functions. Adaptive structures not only tune their properties but also combine contrary properties, and therefore could offer enhanced efficiency, durability, and multi‐functionality; features that make them economically attractive. Despite this, adaptive structures have been put to limited use in everyday life applications. Their use in engineering systems also remains largely restricted to advanced aerospace and space technologies.^[^ ^2^ ^]^ The reason lies in the complexity of their design and implementation. Most existing adaptive structures require sophisticated sensing, feedback, and controls, which make them costly, heavy, and complicated.

In contrast to man‐made designs, natural systems present a striking array of adaptive structures; this is because adaptability has been rewarded throughout evolution. The adaptive response of many natural systems is achieved without active actuation and control in a completely passive way, what we refer to as “automatic” adaptability. The automatic adaptability results from smart design strategies that have reduced the need for complicated active controls in many biological examples. Examples are omnipresent and include, for example, self‐burial behavior of *Erodium cicutarium* seeds,^[^ ^3^, ^4^ ^]^ flexion of scales of seed‐bearing pinecones,^[^ ^5^ ^]^ explosive pollen release of *Kalmia latifolia*,^[^ ^6^ ^]^ passive musculo‐skeletal recovery of cockroach's legs after perturbations,^[^ ^7^ ^]^ and passive redistribution of overlapping flight feathers of birds.^[^ ^8^ ^]^

Insect wings represent a remarkable example for automatic adaptability with significant property and shape changes in flight.^[^ ^9^ ^]^ In contrast to wings of other flying creatures, insect wings lack active flight muscles.^[^ ^10^ ^]^ However, they exhibit large, yet highly controlled passive deformations in flight^[^ ^11^ ^]^ (**Figure** **1**). The adaptability of insect wings is automatic and merely determined by their design architecture. Although wings consist of multiple components that contribute to their adaptability to flight forces, “vein micro‐joints” play a key role in this regard.^[^ ^12^, ^13^, ^14^, ^15^ ^]^ Vein micro‐joints, distributed all over the wings, are compliant joints, formed by the intersection of two or more reinforcing elements, known as veins. In general, they consist of crossing elements and spikes that work as stoppers, which limit deformations and stiffen the wings. The elaborate design of vein joints determines how wings respond to flight forces both locally and globally.

**Figure 1:**
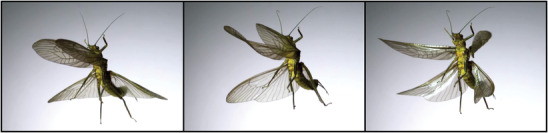
Automatic shape morphing of wings of a stonefly (Plecoptera) during takeoff. Wings undergo significant deformations that are controlled by the wing design. Images are the courtesy of Dr Adrian Smith (Department of Biological Sciences, North Carolina State University).

Recent studies have shown that natural design strategies can be used to develop adaptive structures that automatically respond to external stimuli. These include but are not limited to dragonfly‐inspired flapping kites that adapt their stiffness in response to the wind speed,^[^ ^16^ ^]^ Passive load alleviation of morphing structures for wind turbine blades,^[^ ^17^, ^18^ ^]^ passive morphing of a solar powered flying wing aircraft to enhance solar energy absorbency,^[^ ^19^ ^]^ plant‐inspired composites that reconfigure upon immersion in water or change in the ambient humidity,^[^ ^4^, ^20^ ^]^ pollen‐inspired drug delivery capsules that collapse under pressure,^[^ ^21^, ^22^ ^]^ insect‐inspired artificial fliers that reversibly buckle upon collisions.^[^ ^23^, ^24^, ^25^ ^]^ These studies have opened a new avenue to the design of a new generation of adaptive systems, which can emerge as an independent research area to be called as Mechanical Intelligence (MI).

MI will offer a new paradigm for designing structural components with superior capabilities. It explores nature‐inspired mechanisms for automatic adaptability and applies them to the design of structural components. MI structures can result a drastic change in our surrounding and alter our perception to the design and performance of structural components. MI structures can tune their properties at the right time and right place, and therefore will offer enhanced efficiency, durability, and lifespan. When used in buildings, bridges, and other civil infrastructures, MI structures would prevent catastrophic failures in natural hazards (e.g., earthquakes, hurricanes, and floods), which can have detrimental economic consequences and loss of human lives. MI structures can achieve multi‐functionality; hence they will reduce manufacturing time and cost and require less material and space in comparison to the existing counterparts. In medical applications, MI structures could be used to develop multifunctional surgical instruments to reduce time spent exchanging instruments (10–30% of operation time^[^ ^26^ ^]^), and thereby reduce the safety risk to patients. MI structures have huge potential for helping to overcome global challenges: For example, in the development of adaptive water collection systems that automatically deploy when it rains to collect rainwater; or in our fight against climate change, for example in adaptive wings that can reduce fuel consumption of airplanes and thereby aircraft emissions. MI can further complement other fields, such as embodied intelligence^[^ ^27^ ^]^ and physical artificial intelligence,^[^ ^28^, ^29^ ^]^ which seek to design intelligent systems (mostly robots) either through coupled adaptation of body, brain, and environment or through encoding intelligence into the body independent from the computational intelligence of brain.

In conclusion, MI will open incredible opportunities to engineering design and revolutionize the structures around us, which despite vast technological advances of recent decades are ironically outdated. MI structures, presenting widely accessible solutions for adaptability, will facilitate inclusive and sustainable industrial development, reflective of Goal 9 of the 2030 Agenda for Sustainable Development.^[^ ^30^ ^]^ We anticipate future technological developments and widespread community uptakes for MI.

## Conflict of Interest

The authors declare no conflict of interest.
